# Antigenicity comparison of SARS‐CoV‐2 Omicron sublineages with other variants contained multiple mutations in RBD

**DOI:** 10.1002/mco2.130

**Published:** 2022-04-09

**Authors:** Qianqian Li, Mengyi Zhang, Ziteng Liang, Li Zhang, Xi Wu, Chaoying Yang, Yimeng An, Jincheng Tong, Shuo Liu, Tao Li, Qianqian Cui, Jianhui Nie, Jiajing Wu, Weijin Huang, Youchun Wang

**Affiliations:** ^1^ Division of HIV/AIDS and Sex‐Transmitted Virus Vaccines Institute for Biological Product Control WHO Collaborating Center for Standardization and Evaluation of Biologicals NHC Key Laboratory of Research on Quality and Standardization of Biotech Products and NMPA Key Laboratory for Quality Research and Evaluation of Biological Products National Institutes for Food and Drug Control (NIFDC) Beijing China; ^2^ Jiangsu Recbio Technology Co., Ltd. Taizhou China; ^3^ Graduate School of Peking Union Medical College Beijing China

**Keywords:** BA.1, BA.2, monoclonal antibodies, Omicron sublineages, vaccine, variant immunogen

## Abstract

The severe acute respiratory syndrome coronavirus 2 (SARS‐CoV‐2) variants, particularly those with multiple mutations in receptor‐binding domain (RBD), pose a critical challenge to the efficacy of coronavirus disease 2019 (COVID‐19) vaccines and therapeutic neutralizing monoclonal antibodies (mAbs). Omicron sublineages BA.1, BA.2, BA.3, as well as the recent emergence of C.1.2, B.1.630, B.1.640.1, and B.1.640.2, have multiple mutations in RBD and may lead to severe neutralizing antibody evasion. It is urgent to evaluate the antigenic change of the above seven variants against mAbs and sera from guinea pigs immunized with variants of concern (VOCs) (Alpha, Beta, Gamma, Delta, Omicron) and variants of interest (VOIs) (Lambda, Mu) immunogens. Only seven out of the 24 mAbs showed no reduction in neutralizing activity against BA.1, BA.2, and BA.3. However, among these seven mAbs, the neutralization activity of XGv337 and XGv338 against C.1.2, B.1.630, B.1.640.1, and B.1.640.2 were decreased. Therefore, only five neutralizing mAbs showed no significant change against these seven variants. Using VOCs and VOIs as immunogens, we found that the antigenicity of variants could be divided into three clusters, and each cluster showed similar antigenicity to different immunogens. Among them, D614G, B.1.640.1, and B.1.630 formed a cluster, C.1.2 and B.1.640.2 formed a cluster, and BA.1, BA.2, and BA.3 formed a cluster.

## Graphical abstract

A few mAbs showed strong and broad‐spectrum neutralizing activity against the tested variants. The antigenicity of Omicron sublineages was significantly different from other variants. The Omicron immunogen‐elicited antibody was not sensitive to other variants.

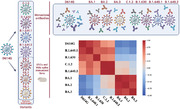

## 1 INTRODUTION

The coronavirus disease 2019 (COVID‐19) pandemic has been going on for more than 2 years.^1^, ^2^ According to the World Health Organization (WHO), more than 440 million confirmed cases have been reported, including more than 5.9 million deaths. The severe acute respiratory syndrome coronavirus 2 (SARS‐CoV‐2), the pathogen of COVID‐19, has evolved and multiple variants have emerged.^3^ Based on the characteristics of the virus, such as its infectivity, the severity of the associated disease, or the impact on vaccines, therapeutics, diagnostics, etc., WHO characterized variants that posed an increased risk to global public health into three categories: variants of concern (VOC), variants of interest (VOI), and variants under monitoring (VUM).^4^ Currently, VOCs include Alpha (B.1.1.7), Beta (B.1.351), Gamma (P.1), Delta (B.1.617.2), and Omicron (B.1.1.529, BA.1). VOIs include Lambda (C.37) and Mu (B.1.621). VUMs change rapidly, with dynamic additions and deletions.^5^

Omicron, discovered in November 2021, attracted immediate and widespread attention because it contains more than 30 mutations in its spike protein.^6^, ^7^ Since then, continued monitoring of Omicron evolution^8^ revealed an increased prevalence of two sublineages, BA.1.1 and BA.2.^9^, ^10^ BA.1.1 has additional R346K mutation compared to BA.1. And in early 2022, BA.2 was becoming more common in a number of countries. By February, BA.2 had become dominant worldwide, overtaking the once‐dominant BA.1.^11^ There were no specific mutations for the BA.3 lineage in the spike protein. Instead, it is a combination of BA.1 and BA.2 spike protein mutations.^12^ At the same time, the new variants, such as C.1.2,^13^, ^14^ B.1.630,^15^ B.1.640.1,^16^ and B.1.640.2,^17^ which defined as VUM in 2021, contain more than three mutations in receptor‐binding domain (RBD). These complicated variants with multiple mutations in RBD have also attracted attention.

Preliminary data from multiple studies suggested that RBD mutations impact the neutralizing activity of monoclonal antibodies (mAbs) and vaccine immune sera.^18^, ^19^ Multiple mutation sites at RBD, especially E484K mutation superimposed other mutations, may lead to breakthrough infection.^20^ At present, the majority of commercial mAbs fail to provide protection against BA.1.^21^ Meanwhile, studies show that BA.1 significantly evades immunity from prior infection or vaccination.^22^, ^23^, ^24^, ^25^ Therefore, the immune evasion of Omicron (BA.1, BA.2, BA.3) and VUMs (C.1.2, B.1.630, B.1.640.1, B.1.640.2), as well as their cross‐reactivity with VOCs and VOIs immunogens, are still open questions. Besides, screening for broad‐spectrum mAbs and developing new generation of vaccines against the complicated variants need further investigation.^26^

In this study, the complicated variants including Omicron sublineages (BA.1, BA.2, BA.3) and VUMs (C.1.2, B.1.630, B.1.640.1, B.1.640.2) are constructed pseudoviruses and their antigenicity is comprehensively analyzed by detecting the neutralizing activity of series mAbs and guinea pig sera immunized by spike protein of VOCs and VOIs. Our results provide important clues for scientists to choose immune strategies against Omicron and future variants.

## 2 RESULTS

### 2.1 The complicated variants with multiple mutations in RBD

With extensive evolution of SARS‐CoV‐2, the more complicated variants may have occurred.^27^ So far, multiple variants with more than three mutations in RBD have been reported. In this study, we focused on seven variants of Omicron sublineages (BA.1, BA.2, BA.3) and the recently emerged VUMs (C.1.2, B.1.630, B.1.640.1, B.1.640.2), which are widely distributed worldwide (Table 1). Omicron sublineage BA.1 contains 37 mutations, among which the N‐terminal domain (NTD) and RBD regions are highly mutated.^8^ The mutations of Omicron sublineage BA.2 is different to BA.1,^9^ especially in the NTD.^12^ Compared with BA.1, S371L, G446S, and G496S are deleted, whereas S371F, T376A, D405N, and R408S are added in the BA.2 RBD.^9^ The RBD of Omicron sublineage BA.3 contains 15 mutation sites, which are highly consistent with BA.1,^12^ while S371F and D405N of BA.3 replace the S371L and G496S mutations of BA.1. The C.1.2 variant, first discovered in South Africa, contains 15 mutations in the spike protein.^13^ The RBD contains three mutations, which are Y449H, E484K, and N501Y. The B.1.630 variant, first discovered in the Dominican Republic, contains 12 mutations in the spike protein, of which the RBD contains three mutations, L452R, T478R, and E484Q.^15^ In December 2021, the B.1.640 variants were found in many countries, and the sequences of its derivatives B.1.640.1 and B.1.640.2 are highly consistent.^17^ The B.1.640.1 variant contains 22 mutations in spike protein, of which the RBD contains five mutations of R346S, N394S, Y449N, F490R, and N501Y. The B.1.640.2 variant contains 23 mutations in the spike protein, and its RBD has additional E484K mutation on the basis of B.1.640.1. Besides, the NTD of B.1.640.1 and B.1.640.2 has a long deletion with a total of nine amino acid deleted (CNDPFLGVY136‐144 Del), which is not common in other variants.

**TABLE 1:** Characteristics of SARS‐CoV‐2 variants

**Classification** **^a^**	**WHO label**	**Pango lineage**	**Mutations in RBD of the spike protein** **^a^**	**Mutations in the rest of the spike protein** **^a^**
VOC	Omicron	B.1.1.529 (BA.1)	G339D, S371L, S373P, S375F, K417N, N440K, G446S, S477N, T478K, E484A, Q493R, G496S, Q498R, N501Y, Y505H	A67V, HV69‐70Del, T95I, G142D, VYY143‐145Del, N211Del, L212I, Ins214EPE, T547K, D614G, H655Y, N679K, P681H, N764K, D796Y, N856K, Q954H, N969K, L981F
		BA.2	G339D, S371F, S373P, S375F,T376A, D405N, R408S, K417N, N440K, S477N, T478K, E484A, Q493R, Q498R, N501Y, Y505H	T19I, LPP24‐26Del, A27S, G142D, V213G, D614G, H655Y, N679K, P681H, N764K, D796Y, Q954H, N969K
		BA.3	G339D, S371F, S373P, S375F, D405N, K417N, N440K, G446S, S477N, T478K, E484A, Q493R, Q498R, N501Y, Y505H	A67V, HV69‐70Del, T95I, G142D, VYY143‐145Del, N211Del, L212I, D614G, H655Y, N679K, P681H, N764K, D796Y, Q954H, N969K
	Alpha	B.1.1.7	N501Y	HV69‐70Del, Y144Del, A570D, D614G, P681H, T716I, S982A, D1118H
	Beta	B.1.351	K417N, E484K, N501Y	L18F, D80A, D215G, LAL242‐244Del, D614G, A701V
	Gamma	P.1	K417T, E484K, N501Y	L18F, T20N, P26S, D138Y, R190S, D614G, H655Y, T1027I, V1176F
	Delta	B.1.617.2	L452R, T478K	T19R, G142D, EF156‐157Del, R158G, D614G, P681R, D950N
VOI	Lambda	C.37	L452Q, F490S	G75V, T76I, RSYLTPG246‐252Del, D253N, D614G, T859N
	Mu	B.1.621	R346K, E484K, N501Y	T95I, Y144S, Y145N, D614G, P681H, D950N
VUM		C.1.2	Y449H, E484K, N501Y	P9L, C136F, Y144Del, R190S, D215G, AL243‐244Del, D614G, H655Y, N679K, T716I, T859N
		B.1.630	L452R, T478R, E484Q	P9L, C136F, Y144Del, A222V, AL243‐244Del, D614G, H655Y, D950N
		B.1.640.1	R346S, N394S, Y449N, F490R, N501Y	P9L, E96Q, CNDPFLGVY136‐144 Del, R190S, I210T, D614G, P681H, T859N, D936H
		B.1.640.2	R346S, N394S, Y449N, E484K, F490S, N501Y	P9L, E96Q, CNDPFLGVY136‐144 Del, R190S, D215H, D614G, P681H, T859N, D1139H

*Note*: The spike protein mutations of the SARS‐CoV‐2 variants used in this study are listed.

Abbreviations: RBD, receptor‐binding domain; VOC, variants of concern; VOI, variants of interest; VUM, variants under monitoring.

^a^ The classification of variant strains is regularly adjusted based on the SARS‐CoV‐2 continuous evolution and spread, as well as a better understanding of the impact of the variants. As a result, the classification of variants is dynamic, constantly being removed or added.

^b^ The mutation sites of the variants represent the characteristics of most sequences, and individual mutation sites can only be detected in partial sequences, which are not shown here.

### 2.2 The complicated variants with multiple mutations in RBD evade neutralization of mAbs

**FIGURE 1:**
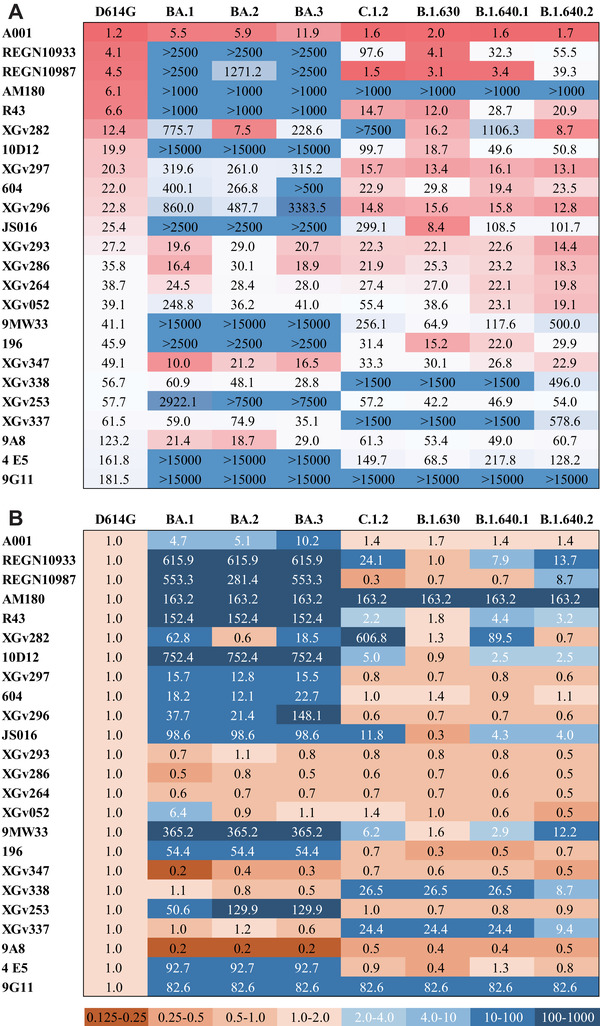
Antigenicity analysis of variants to a panel of monoclonal antibodies (mAbs). (A) Heatmap of the neutralizing activity of 24 mAbs derived from infected or vaccine immunized persons against D614G and seven variant pseudoviruses. EC50 data of mAbs are results from three independent experiments. Darker red indicates better neutralizing activity of the mAbs and lower EC50 value. Conversely, darker blue indicates worse neutralizing activity and higher EC50 values. EC50, median effect concentration. Also see in Figure 2. (B) The heatmap represents the ratio of EC50 values between seven variants and D614G reference. Brown or blue in the scale bar indicate increased or decreased sensitivity of the pseudovirus to mAbs, respectively. Darker brown indicates higher neutralizing activity of mAbs against the variant compared with D614G. In contrast, darker blue indicates lower neutralizing activity of mAbs against the variant compared with D614G

**FIGURE 2:**
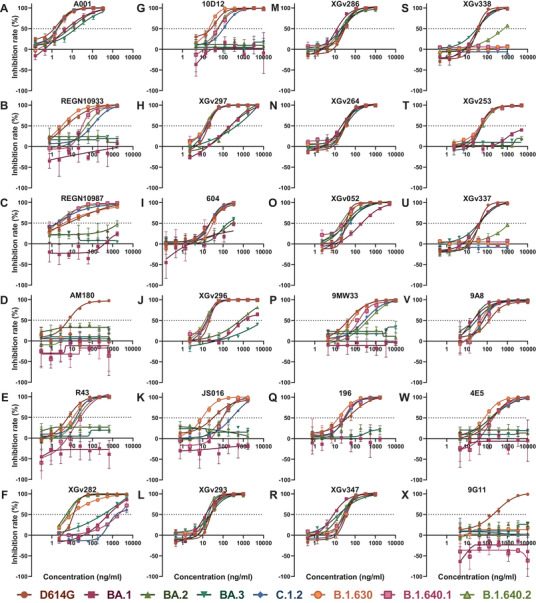
Neutralization curves of monoclonal antibodies (mAbs) against variants. Neutralization curves of 24 mAbs against D614G and seven variant pseudoviruses. The inhibition rate at different mAb concentrations was calculated, and then the neutralization curve data were drawn using GraphPad software. All data are the result of three replicates. The *x*‐axis is the antibody concentration, the *y*‐axis represents the inhibition rate of different pseudoviruses, and the dotted line represents the 50% inhibition rate. (A–S) The mAbs A001, REGN10933, REGN10987, AM180, R43, XGv282, 10D12, XGv297, 604, XGv296, JS016, XGv293, XGv286, XGv264, XGv052, 9MW33, 196, XGv347, XGv338, XGv253, XGv337, 9A8, 4E5, and 9G11, respectively. Related to Figure 1

Neutralization assay was performed using pseudoviruses carrying D614G, Omicron sublineages (BA.1, BA.2, BA.3), and VUMs (C.1.2, B.1.630, B.1.640.1, B.1.640.2) spike proteins. Neutralizing activity of 24 mAbs from different sources was analyzed (Figure 1A). The EC50 of these 24 mAbs against D614G pseudoviruses range from 1.2 to 181.5 ng/ml, indicating that all mAbs were particularly effective against D614G. The neutralizing activity of mAbs against variants was compared to that against D614G (Figures 1B and 2). The results showed that Omicron escaped the neutralization of most mAbs, with 17, 15, and 16 mAbs reducing the neutralization activity by more than four‐fold against Omicron sublineages. Among them, 11 mAbs were reduced by >50‐fold against Omicron (Figures 1B and 2). The mAbs A001, XGv293, XGv286, XGv264, XGv347, XGv338, XGv337, and 9A8 had high neutralizing activity against Omicron sublineages, while the therapeutic mAb 604 showed moderate neutralizing activity. The mAbs such as REGN10933, REGN10987, JS016 almost lost their neutralizing effect on Omicron. In addition, XGv282 and XGv052 have lineage‐specific neutralizing activities. The neutralizing activity of XGv282 against BA.1 and BA.3 was decreased by 62.8‐ and 18.5‐fold, but no change against BA.2. The neutralizing activity of XGv052 was only reduced 6.4‐fold for BA.1, but not for BA.2 and BA.3. Besides, XGv347 and 9A8 had more robust neutralization protection against BA.1, BA.2, and BA.3.

In addition, most of the mAbs showed high neutralizing activity against the emerged VUMs, with 16, 20, 16, and 17 mAbs against C.1.2, B.1.630, B.1.640.1, and B.1.640.2 with EC50 <100 ng/ml, respectively (Figure 1A). But nine, four, eight, and eight mAbs reduced the neutralization activity by more than four‐fold against C.1.2, B.1.630, B.1.640.1, and B.1.640.2, respectively (Figures 1B and 2). Among them, AM180 and 9G11 showed >50‐fold decreased, and XGv338 and XGv337 showed >10‐fold decreased neutralization activity against these four variants. Neutralizing activity of mAb XGv282 was decreased by 606.8‐ and 89.5‐fold against C.1.2 and B.1.640.1, respectively, but the neutralizing activity against B.1.630 and B.1.640.2 was consistent with D614G. In addition, mAbs REGN10933, REGN10987, and JS016 showed varying degrees of weakened neutralization protection against VUMs. Among them, mAb REGN10933 exhibited 24.1‐, 7.9‐, and 13.7‐fold decreases in neutralizing activity against C.1.2, B.1.640.1, and B.1.640.2. The mAb REGN10987 exhibited 8.7‐fold decrease in neutralizing activity against B.1.640.2. And mAb JS016 exhibited 11.8‐, 4.3‐, and 4.0‐fold decreases in neutralizing activity against C.1.2, B.1.640.1, and B.1.640.2, respectively.

These results indicated that a few mAbs have high neutralizing activity against Omicron variants, whereas most mAbs have strong neutralizing activity against emerging VUMs. In conclusion, only seven mAbs did not significantly reduce the neutralizing activity of BA.1, BA.2, and BA.3, whereas XGv337 and XGv338 among these seven mAbs exhibited decreased neutralizing activity against C.1.2, B.1.630, B.1.640.1, and B.1.640.2. Therefore, only mAbs XGv293, XGv286, XGv264, XGv347, and 9A8 showed no significant change in neutralizing activity against the seven variants.

### 2.3 Antigenicity assessment of the complicated variants with multiple RBD mutations against different immunogenic sera

**FIGURE 3:**
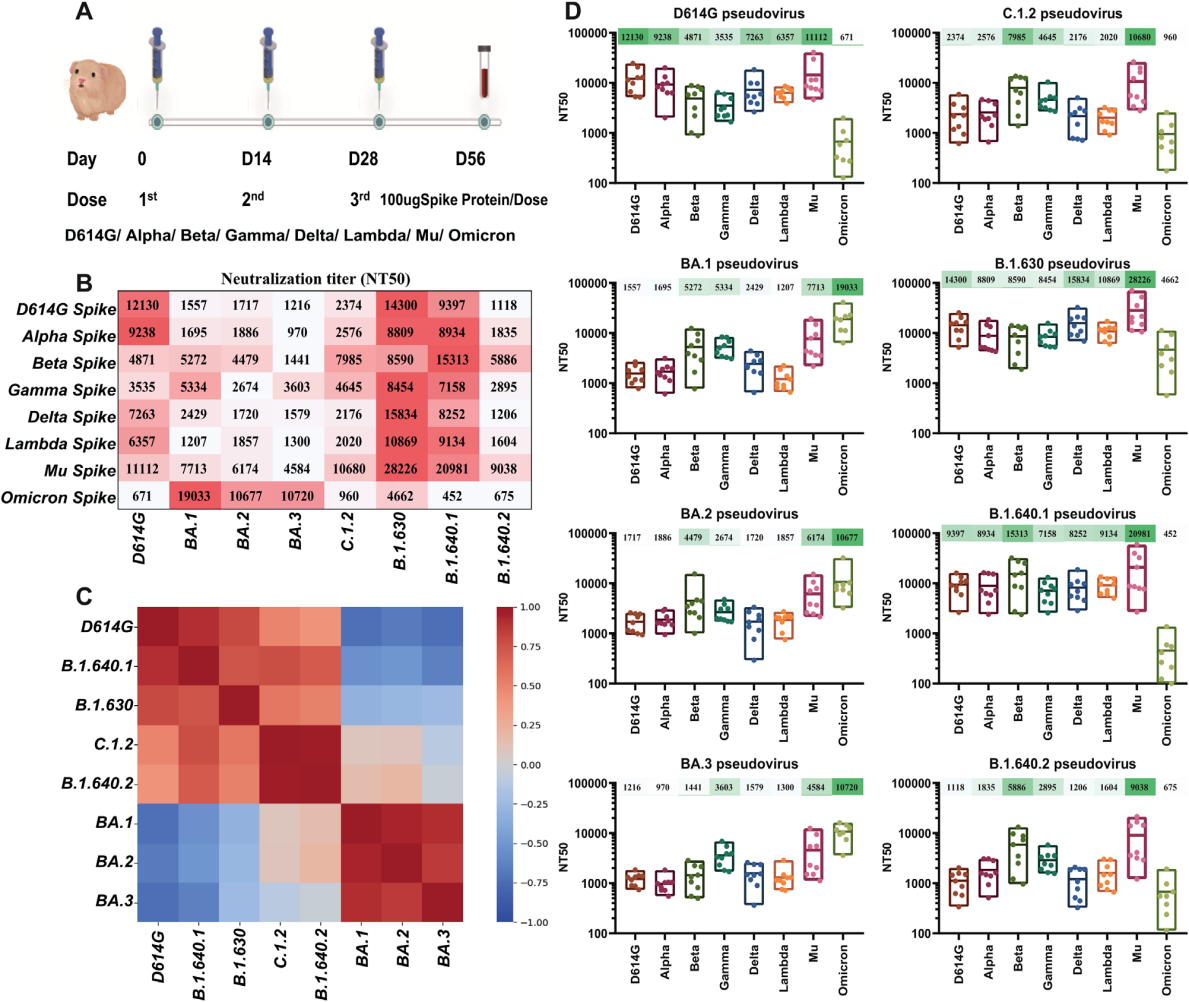
Antigenicity analysis of Omicron sublineages and variants under monitoring (VUMs) against variants of concern (VOCs) and variants of interest (VOIs) spike protein immunized sera. (A) Schematic diagram of the procedures of vaccine immunization and blood collection. Nine guinea pigs were subcutaneously immunized with 100 μg of spike protein and alum adjuvant mixed, and immunized three times on D0, D14, and D28, respectively. Serum was collected 28 days after the third immunization. (B) Neutralizing activity of guinea pig immune sera against variants. The *y*‐axis represents guinea pig sera immunized with different immunogens, and the *x*‐axis represents different variant pseudoviruses. Values are the mean values of NT50 of eight to nine sera of different immunogens against variants. Three replicate experiments were performed for each serum. Darker red indicates better neutralizing activity of the serum against the variant and the higher NT50 value. NT50, 50% neutralizing titer. (C) Heatmap of Spearman correlation coefficient between different variants. The NT50 values of different variants to different immunogen immune sera were transformed by logarithmic scale, assembled into an 8 × 8 matrix and subjected to principal component analysis. The Spearman correlation coefficient (*r* ^2^) matrix between each variant is shown in the form of heatmap. The scale bar represents the correlation coefficient. Dark red indicates a positive correlation between different immunogens, and dark blue indicates a negative correlation. (D) Results of each virus against multiple immunogenic sera. The antigenic performance of each variant is presented individually and the NT50 shown as the mean and its range. The *x*‐axis represents the sera immunized with eight spike protein immunogens, and the *y*‐axis represents the NT50 value. Each point represents the NT50 of three replicates of each serum. The mean NT50 of sera from eight to nine guinea pigs is marked above the corresponding variant

To evaluate the antigenicity of the complicated variants with multiple RBD mutations, we immunized guinea pigs with spike trimeric proteins of D614G, VOCs (Alpha, Beta, Gamma, Delta, Omicron), and VOIs (Lambda, Mu). Serum samples were collected 4 weeks after the third immunization. Then, eight pseudoviruses, D614G, Omicron (BA.1, BA.2, BA.3), and VUMs (C.1.2, B.1.630, B.1.640.1, B.1.640.2) were used to evaluate the difference of antibodies in sera immunized with different VOCs and VOIs (Figure 3A).

D614G original virus can be well neutralized by D614G, VOCs (Alpha, Beta, Gamma, Delta), and VOIs (Lambda, Mu) spike protein immunized sera, with NT50 values of 12,130, 9238, 4871, 3535, 7263, 6357, and 11,112, respectively (Figure 3B,D). Among them, the D614G immunized serum had the strongest neutralization protection to the original D614G strain. While the D614G virus escaped the Omicron spike‐immunized sera, the NT50 was only 671. The B.1.640.1 variant can be well neutralized by spike protein immunized serum, and the NT50 were all above 7158. While B.1.640.1 variant escaped the Omicron spike‐immunized sera, the NT50 was only 452 (Figure 3B,D). Seven groups of sera can well neutralize the B.1.630 variant, and the NT50 is all above 8454. Although slightly resistant to Omicron spike‐immunized sera, the decrease was within four‐fold (NT50 = 4662) and was still well neutralized (Figure 3B,D). Besides, the sera immunized with spike protein from D614G, Alpha, Delta, and Mu variants are more protective against D614G, B.1.640.1, and B.1.630 variants. Cluster analysis showed that D614G, B.1.640.1, and B.1.630 showed similar antigenicity to different immunogens (Figure 3C).

The antigenicity of C.1.2 variant against these eight groups of sera can be divided into three categories (Figure 3B,D). The C.1.2 variant was well neutralized by Beta, Gamma, and Mu spike‐immunized sera, with NT50 of 7985, 4645, and 10,680, respectively. The C.1.2 variant showed slight resistance to D614G, Alpha, Delta, and Lambda spike‐immunized sera with NT50 between 2020 and 2576. The C.1.2 variant escaped from Omicron spike‐immunized sera with NT50 of only 960. The antigenicity of B.1.640.2 variant to these eight groups of sera was similar to that of C.1.2 variant, but the neutralization titer against B.1.640.2 variant was weaker than that against C.1.2 (Figure 3B,D). Cluster analysis showed that C.1.2 and B.1.640.2 variant showed similar antigenicity to different immunogens (Figure 3C).

BA.1, BA.2, and BA.3 variants can be neutralized by Omicron spike‐immunized sera with NT50 values all above 10,000 (Figure 3B,D). They could also be neutralized by Mu spike‐immunized serum with NT50 values above 4584. The BA.1, BA.2, and BA.3 variants showed slight resistance to Beta and Gamma spike‐immunized sera, especially the NT50 value of BA.2 variant against Gamma spike‐immunized sera was only 2674, and BA.3 variant against Beta spike‐immunized sera was only 1441. In addition, BA.1, BA.2, and BA.3 variants were strongly resistant to D614G, Alpha, Delta, and Lambda spike‐immunized sera, with NT50 values ranging from 970 to 2429. Cluster analysis showed that BA.1, BA.2, and BA.3 variants showed similar antigenicity to different immunogens (Figure 3C).

## 3 DISCUSSION

Since the outbreak of COVID‐19, the SARS‐CoV‐2 virus has evolved multiple variants. Recently, the Omicron sublineages BA.1, BA.1.1, BA.2, and BA.3 spread rapidly in various countries, becoming the main epidemic variants and classified as VOC by WHO.^28^ In addition, C.1.2, B.1.630, B.1.640.1, and B.1.640.2 variants also appeared in various places and were classified as VUM by WHO.^14^, ^16^ The spike proteins of these variants have a large number of mutations, especially the RBDs have more than three mutations. There is evidence that these variants can enhance the transmissibility, fitness, infectivity of the virus, and reduce the protective efficacy of vaccines and therapeutic antibodies, which quickly raised unprecedented concerns.^6^, ^7^, ^29^ At present, the results showed that BA.1 can largely escape vaccination, convalescent serum, and most approved mAbs.^21^, ^22^, ^30^, ^31^ In response to Omicron, researchers quickly screened out neutralizing mAbs and specific vaccines against BA.1.^32^, ^33^ But it is not clear whether these mAbs can protect against the rapidly developing BA.2 and other variants. Moreover, it is unclear whether the BA.1‐specific vaccine has broad‐spectrum neutralizing effect against other variants. In addition, there are few research results on BA.2, BA.3, C.1.2, B.1.630, B.1.640.1, and B.1.640.2 variants. At present, the antigenic changes of these variants to mAbs and vaccines are not known.

In this study, we first tested the neutralizing activity of 24 mAbs derived from infected individuals or vaccine immune screening against seven variants. These include not only therapeutic mAbs already on the market and those under development, but also 11 mAbs derived from memory B cells in three‐dose vaccinees. Our data showed that the vast majority of mAbs’ neutralizing activity against BA.1, BA.2, and BA.3 variants was reduced or even lost. Among the 24 mAbs we tested, 22 mAbs showed consistent response against BA.1, BA.2, and BA.3, and two mAbs were inconsistent, which may be related to the individual amino acid substitution in RBD. The RBD of C.1.2, B.1.630, B.1.640.1, and B.1.640.2 variants contain three to six mutations. Although some mAbs have reduced neutralizing activity, a considerable number of mAbs retain their neutralization activity against these variants. The approved neutralizing antibody drugs REGN10933 and REGN10987 almost lost protection against Omicron sublineages, which is consistent with previous reports.^21^, ^34^ Meanwhile, the neutralizing activity of REGN10933 against C.1.2, B.1.640.1, and B.1.640.2 variants was decreased, and the neutralizing activity of REGN10987 against B.1.640.2 variant was decreased. It is speculated that it is related to the K417 and E484 mutations. Therefore, mAbs REGN10933 and REGN10987 are not suitable for the treatment of variants with multiple RBD mutations. The neutralizing activity of the approved mAb 196 against Omicron sublineages also decreased by more than 50‐fold, suggesting the need for further development of new therapeutic mAbs.^35^ In addition, mAbs JS016^36^ and 9MW33^37^ under development almost lost their protection against Omicron sublineages, and also had slightly decreased neutralizing activity against C.1.2, B.1.640.1, and B.1.640.2 variants. The neutralizing activity of mAb JS016 was reduced due to the N501Y mutation in the variants. The neutralizing activity of 604 against Omicron sublineages was reduced, which was consistent with the previous reports.^21^ AM180 and 9G11 lost neutralizing activity to the other seven variants except for D614G, presumably due to the E484 mutation.^38^ Monoclonal antibody 10D12 almost lost protection against Omicron sublineages, presumably related to the K417N mutation, which is consistent with the previous reports.^38^ The neutralization protection of mAb A001 against Omicron sublineages decreased, but the EC50 values against the eight pseudoviruses tested ranged from 1.2 to 11.9 ng/ml. Among the 24 mAbs we tested, A001 showed the best neutralizing activity against the variants, suggesting that this mAb has the potential to be a neutralizing antibody drug for the treatment of COVID‐19. Monoclonal antibody 9A8 showed broad‐spectrum neutralizing activity, and the neutralizing activity against Omicron sublineages was increased.

Eleven mAbs isolated from memory B cells of three‐dose vaccinees had different neutralizing properties.^32^ The neutralizing activity of mAbs XGv296, XGv297, and XGv253 against Omicron sublineages decreased by more than 10‐fold, but retained the neutralization activity against C.1.2, B.1.630, B.1.640.1, and B.1.640.2 variants. XGv337 and XGv338 showed good neutralization protection against Omicron sublineages, but the protection against C.1.2, B.1.630, B.1.640.1, and B.1.640.2 variants was reduced by more than eight‐fold, presumably related to Y449 and L452 mutations. The neutralizing activity of mAb XGv282 against BA.1 and BA.3 was decreased by 62.8‐ and 18.5‐fold, but there was no significant change against BA.2. Structural analysis showed that the antigenic binding epitope of XGv282 was located at the right shoulder of RBD, whereas the G446S mutation of BA.1 and BA.3 affected the binding of the mAb.^32^ In addition, the neutralization activity of XGv282 against C.1.2 decreased by 606.8‐fold, which was speculated to be related to the Y449H mutation of C.1.2 variant. Surprisingly, the neutralizing activity of XGv282 against B.1.640.1 was reduced by 89.5‐fold, but no significant change against B.1.640.2, presumably due to the additional E484K mutation altering the spatial conformation of the spike protein. The neutralizing activity of XGv052 against BA.1 variant was reduced by 6.4‐fold, but the neutralizing activity against BA.2 and BA.3 did not change. It was speculated that the BA.1 variant RBD contained G446S and G496S, whereas the BA.2 and BA.3 variants did not contain these two mutations, thus leading to this result. The mAbs XGv264, XGv286, XGv293, and XGv347 exhibited broad‐spectrum neutralizing activity against the eight pseudoviruses tested. Surprisingly, the neutralizing activity of XGv347 against Omicron sublineages was not reduced, but enhanced. In previous literature, its structure was analyzed and it was found that the XGv347‐Omicron S complex structures had three distinct conformational states. Moreover, XGv347 binds to an epitope at RBD, largely overlapping with the patch targeted by ACE2. And this epitope is conserved among different SARS‐CoV‐2 variants, resulting in the broad‐spectrum of XGv347 to VOCs and VUMs.

In this study, the recombinant spike trimer protein was used as an immunogen to simulate vaccination or natural infection. The antigenicity analysis of variants showed that the variants could be divided into three clusters, and each cluster showed similar antigenicity to different immunogens. Among them, D614G, B.1.640.1, and B.1.630 formed a cluster, C.1.2 and B.1.640.2 formed a cluster, and BA.1, BA.2, and BA.3 formed a cluster. However, it has been documented that although BA.1 and BA.2 both evaded the vaccine‐induced antibody response, this phenomenon resulted from different antigenic characteristics.^39^ Thus, it is speculated that Omicron BA.1 and BA.2 are antigenically distinct SARS‐CoV‐2 variants.^39^ But there is no doubt that both the immunogenicity and antigenicity of Omicron have evolved into a relatively distant branch compared to other variants. This is consistent with clinical manifestations of Omicron in the real world. It has been reported that after immunization with inactivated vaccine, recombinant protein vaccine and mRNA vaccine, the antibody level decreases with the extension of inoculation time, leading to the increase of breakthrough infection of Omicron.^18^, ^22^

Our data indicated that D614G, Alpha, Delta, and Mu were similar in immunogenicity, and these sera had significantly reduced protection against Omicron sublineages (Figure S1). Surprisingly, these four groups of sera also had poor neutralizing activity against C.1.2 and B.1.640.2, which may be caused by E484K mutation. The strong neutralization protection of Delta spike‐immunized sera against B.1.630 may be due to the L452R mutation contained in the Delta immunogen. Similarly, the strong neutralization protection of Lambda spike‐immunized sera against B.1.630 and B.1.640.1 may be due to the presence of L452Q (B.1.630 containing L452R mutation) and F490S (B.1.640.1 containing F490R mutation but not E484K mutation) in the Lambda immunogen. The immunogenicity of Beta, Gamma, and Mu were similar, probably because these three immunogens all contained E484K and N501Y mutations (Figure S1). Surprisingly, these immunogens exhibited protection against Omicron. Before the outbreak of Omicron, several vaccine companies had developed a new generation of vaccines.^40^ The mRNA vaccine and recombinant protein vaccine against the Beta variant are already in the clinical studies.^41^, ^42^ Therefore, it is speculated that the Beta vaccine booster can achieve partial resistance to the Omicron variants. Additionally, the Mu immunogen can induce high neutralizing protective antibodies against a broad spectrum of variants, which is also worthy of further study (Figure S1). Besides, we found significant differences in immunogenicity between Omicron and these seven variants. Omicron spike‐immunized sera can well neutralize the BA.1, BA.2, BA.3 variants (Figure S1). This is consistent with the results of Omicron‐specific mRNA vaccine, the antibodies produced by the Omicron vaccine immunization can achieve protection against Omicron variants.^33^, ^43^ In addition, Omicron spike‐immunized sera also has a certain protective effect on B.1.630, possibly due to Omicron immunogen containing T478K and E484A. However, Omicron‐immunized sera was extremely poorly protected against the other variants. This suggests that natural infection with Omicron and specific vaccines developed against Omicron may not provide useful or broad herd immunity against other variants. Therefore, the Omicron‐specific vaccine can be used as a booster vaccine, but it cannot replace the original vaccine.

The limitation of this study is that we only used pseudotyped viruses and did not use live viruses for validation. However, it is worth noting that pseudoviruses are now widely used in virus research and vaccine evaluation, and have been shown to be well correlated with authentic viruses.^39^ At the same time, most of the mAbs we tested are still in preclinical studies, and only a few mAbs have been used in clinical practice. In addition, in order to compare the differences between Omicron sublineages, we selected some mAbs that are known to protect the BA.1 variant, which may partially conceal the fact that Omicron has a huge challenge to existing mAbs. Besides, we only used sera from guinea pigs and did not test sera from humans. This is because it is difficult to obtain human sera cohorts immunized or infected with VOCs and VOIs variants in the same population background, at the same dose, and at the same time interval. Therefore, in order to accurately compare cross‐immune responses between all significant variants, animal experiments are the fastest and optimal option.

Taken together, our study shows that a few mAbs have strong and broad‐spectrum neutralizing activity against the tested variants. People vaccinated with the original strain or infected with Alpha, Delta, and Lambda may not resist to Omicron infection. At the same time, Omicron‐elicited antibodies were also insufficient to protect against the other variants. In addition, there were differences in the antigenicity of variants. These antigenicity differences were mainly caused by mutations in the RBD. These results suggest that E484, N501, and other sites may be important to be considered in the development of the next‐generation vaccines.

## 4 METHODS AND MATERIALS

### 4.1 Cells

293T (American Type Culture Collection [ATCC], CRL‐3216) and Huh‐7 (Japanese Collection of Research Bioresources [JCRB], 0403) were cultured using Dulbecco's modified Eagle medium (DMEM, high glucose; Hyclone) supplied with 100 U/ml of penicillin‐streptomycin solution (Gibco) and 10% fetal bovine serum (FBS, Pansera ES, PAN‐Biotech) in a 5% CO_2_ environment at 37°C. Cells were passaged every 2–4 days using 0.25% Trypsin‐EDTA (Gibco).

### 4.2 Plasmids

D614G (GISAID: EPI_ISL_766872), BA.1 (GISAID: EPI_ISL_6590782.2), BA.2 (GISAID: EPI_ISL_7644798), BA.3 (GISAID: EPI_ISL_7740765), C.1.2 (GISAID: EPI_ISL_8801147), B.1.630 (GISAID: EPI_ISL_6368831), B.1.640.1 (GISAID: EPI_ISL_8013598), and B.1.640.2 (GISAID: EPI_ISL_8376567) spike protein expression plasmids are all entrusted to General Biology (Anhui) Co., Ltd. The spike protein nucleotide sequences were optimized using a mammalian codon, and ligated to the eukaryotic expression vector pcDNA3.1 through *Bam*HI and *Xho*I.

### 4.3 Monoclonal antibodies

Twenty‐four anti‐SARS‐CoV‐2 spike mAbs were used in this study. The mAb sources were as follows: mAbs A001 and AM180 were from Acro Biosystems Co.; mAbs 10D12, 9G11, 9A8, and 4E5 were from Dr. Yuelei Shen of Beijing Biocytogen Inc.; mAb 9MW33 was from Mabwell Bioscience Co.; mAb 604 was from Prof. Sunney Xie of Peking University; mAb JS016 was provided by Prof. Jinghua Yan from the Institute of Microbiology, Chinese Academy of Sciences; mAb R43 was from Prof. Yongjun Guan; mAb 196 acquired from Prof. Linqi Zhang of Tsinghua University; mAbs REGN10933 and REGN10987 were developed by Regeneron; and mAbs XGv282, XGv297, XGv296, XGv293, XGv286, XGv264, XGv052, XGv347, XGv338, XGv253, and XGv337 were given by Prof. Xiangxi Wang, Institute of Biophysics, Chinese Academy of Sciences.

### 4.4 Sera from guinea pigs immunized with SARS‐CoV‐2 variant spike protein

Female guinea pigs (bodyweight 200–220 g) were used as experimental animals, and divided into eight groups with nine animals in each group. Each guinea pig was subcutaneously immunized with 100 μg of purified spike proteins of different SARS‐CoV‐2 variants (Acro Biosystems Co.), including the D614G reference strain, current VOCs (Alpha, Beta, Gamma, Delta, Omicron) and VOIs (Lambda, Mu). Spike protein (100 μg) was mixed with alum adjuvant and immunized once every 14 days for three inoculations in total. Sera were collected 28 days after the third immunization for subsequent experiments.

### 4.5 Construction and titration of SARS‐CoV‐2 pseudotyped viruses

The pseudotyped viruses bearing the spike protein were generated and titrated as previously described. Briefly, 293T cells were first transfected with the SARS‐CoV‐2 spike protein expression plasmids of D614G or VOCs/VOIs (Alpha, Beta, Gamma, Delta, Lambda, Mu, and Omicron). The transfected 293T cells were simultaneously infected with VSV pseudotyped virus, G*ΔG‐VSV (Kerafast, Boston, MA). After 6 h of incubation, cells were washed twice with PBS before complete culture medium was added. Pseudovirus‐containing supernatants were harvested after 24 and 48 h, and stored at −80°C for future use. Titrations of SARS‐CoV‐2 pseudotyped viruses were assessed by infecting Huh‐7 cells with three‐fold serial dilutions. The cell culture plate was incubated at 37°C with 5% CO_2_ for 24 h. Chemiluminescent signals were detected according to the protocol of the Britelite plus reporter gene assay system (PerkinElmer, Waltham, MA).

### 4.6 In vitro neutralization assay with pseudotyped viruses

For the in vitro pseudotyped virus neutralization assays, plasma samples (starting at 1:30) or antibodies (appropriate concentrations) were serially diluted and mixed with 1.3 × 10^4^ TCID_50_ of pseudotyped viruses in 96‐well plates at 37°C for 1 h. It was then mixed with Huh‐7 cells (20,000/wells) and subsequently incubated for 24 h. As previously described, the luciferase luminescence (RLU) of each well was measured, and the 50% neutralization titer (NT50) was calculated using the Reed–Muench method to evaluate the neutralizing antibody content in the samples.

### 4.7 Spearman's correlation coefficient heatmap

The NT50 values corresponding to each serum/virus were log scale transformed, assembled into an 8 × 8 matrix. A matrix of Spearman's correlation coefficients (*r* ^2^) between each virus strain is displayed in the form of a heatmap.

### 4.8 Software and data analysis

Figures were generated using GraphPad Prism 8 software (GraphPad, San Diego, CA, USA) and Microsoft Excel. Values are shown as means with range. The least squares fit of Abs is calculated by sigmoidal 4PL (where *X* is log concentration) standard curve. Spearman's correlation coefficient (*r* ^2^) heatmap of virus strain was generated using Python language (https://www.python.org).

## CONFLICT OF INTEREST

Qianqian Li is an employee of Jiangsu Recbio Technology Co., Ltd. All other authors declare that there is no conflict of interest.

## ETHICS APPROVAL

Guinea pigs were handled under institutional (NIFDC, Beijing, China) guidelines for laboratory animal care and use, and the animal study protocol was approved by the Animal Care and Use Committee of the NIFDC.

## Supporting information

Figure S1 The immunogenicity of D614G, VOCs, and VOIs is different. (A) Heatmap of Spearman correlation coefficient between different immunogens. The NT50 values corresponding to the immunogen immunized serum neutralized variants were transformed by logarithmic scale, assembled into an 8 × 8 matrix, and subjected to principal component analysis. The Spearman correlation coefficient (*r* ^2^) matrix between each immunogen is shown in the form of heatmap. (B–I) Results of each immunogen against multiple viruses. Results for sera immunized with each immunogen are presented separately and NT50 shown as the mean and its range. The *x*‐axis represents the eight SARS‐CoV‐2 variants, and the *y*‐axis represents the NT50 value. Each point represents the NT50 of three replicates of each serum. The mean NT50 of sera from eight to nine guinea pigs is marked above the corresponding variant.

## Data Availability

All data generated or analyzed during this study are available from the corresponding author upon reasonable request.
