# Supplementary figures and images for: Antigenicity comparison of SARS‐CoV‐2 Omicron sublineages with other variants contained multiple mutations in RBD
Source: MedComm (2020). 2022 Apr 9;3(2):e130. doi: 10.1002/mco2.130 (PMC8994617; doi:10.1002/mco2.130)

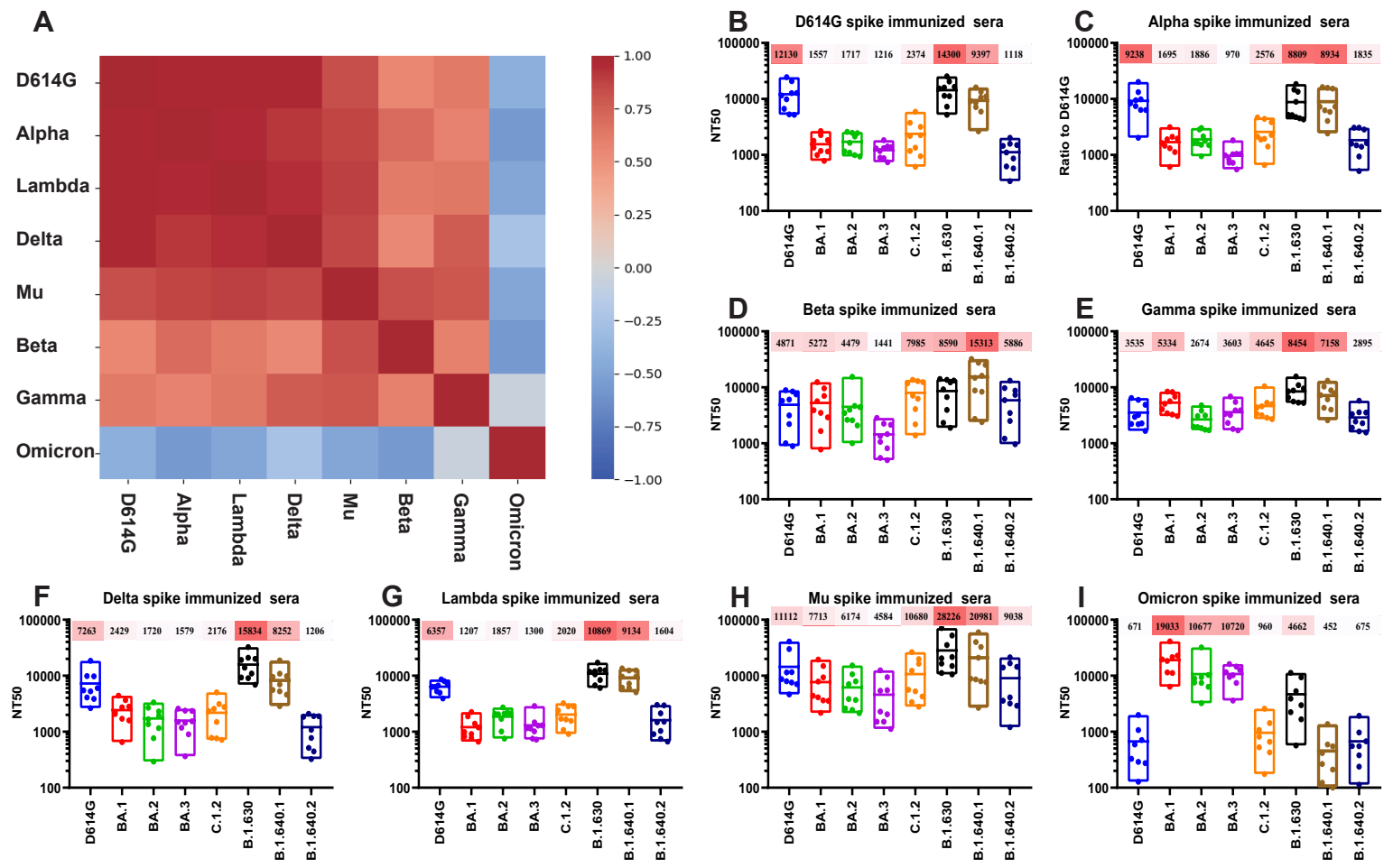

Supplement: Supplementary file 1 — Figure S1 The immunogenicity of D614G, VOCs, and VOIs is different. (A) Heatmap of Spearman correlation coefficient between different immunogens. The NT50 values corresponding to the immunogen immunized serum neutralized variants were transformed by logarithmic scale, assembled into an 8 × 8 matrix, and subjected to principal component analysis. The Spearman correlation coefficient (r 2) matrix between each immunogen is shown in the form of heatmap. (B–I) Results of each immunogen against multiple viruses. Results for sera immunized with each immunogen are presented separately and NT50 shown as the mean and its range. The x‐axis represents the eight SARS‐CoV‐2 variants, and the y‐axis represents the NT50 value. Each point represents the NT50 of three replicates of each serum. The mean NT50 of sera from eight to nine guinea pigs is marked above the corresponding variant. [file MCO2-3-0-s001.pdf]
